# Profiles of cytokines in patients with antineutrophil cytoplasmic antibody-associated vasculitis

**DOI:** 10.3389/fimmu.2024.1428044

**Published:** 2024-07-23

**Authors:** Weiwei Hao, Wei Li, Xiaoying Wang, Fang Dong, Peiling Liu, Xin Zhang, Rui Liu, Tianfang Li, Lei Zhang, Shengyun Liu

**Affiliations:** Department of Rheumatology, The First Affiliated Hospital of Zhengzhou University, Zhengzhou, Henan, China

**Keywords:** ANCA-associated vasculitis, biomarker, disease activity, signaling pathway, cytokine panel

## Abstract

**Objective:**

This study aimed to identify plasma biomarkers that are significantly altered in patients with antineutrophil cytoplasmic antibody (ANCA)-associated vasculitis (AAV) and are closely associated with AAV disease activity, as well as to explore their role in the pathogenesis of AAV.

**Methods:**

Cytokines were measured using Human Immune Response Panel 80-Plex in plasma from 59 patients with AAV and 20 healthy controls (HCs). The differentially expressed cytokines between the two groups and the possible signaling pathway involved in the pathogenesis of AAV were analyzed by bioinformatics. Relationship analysis was performed between these cytokines and clinical parameters to identify the biomarkers that can effectively indicate disease activity.

**Results:**

We identified 65 differentially expressed cytokines between the two groups. Among them, 43 cytokines significantly affected the risk of AAV. Bioinformatic analysis showed that the 43 cytokines were primarily enriched in signaling pathways such as cytokine-cytokine receptor interaction, viral protein interaction with cytokine and cytokine receptor, chemokine signaling pathway, and IL-17 signaling pathway. The levels of 25 cytokines were significantly positively correlated with Birmingham Vasculitis Activity Score (BVAS), and the levels of 2 cytokines were significantly negatively correlated with BVAS. Receiver operating characteristic analysis showed that 9 cytokines can distinguish between disease relapse and remission (PTX3: area under curve (AUC)=0.932, IL34: AUC=0.856, IL2RA: AUC=0.833, CCL23: AUC=0.826, VEGFA: AUC=0.811, TNFSF13: AUC=0.795, Granzyme A: AUC=0.788, CSF3: AUC=0.773 and IL1A: AUC=0.765). The elevated levels of these 9 cytokines suggested a risk of disease relapse. The AUC of CCL11 in disease relapse and remission was 0.811 (*p*=0.0116). Unlike the other 9 cytokines, a negatively association existed between CCL11 level and the risk of disease relapse.

**Conclusion:**

A group of cytokines that may be involved in AAV pathogenesis was identified. Increased PTX3, IL34, IL2RA, CCL23, and VEGFA levels correlate with active disease in AAV and may be used as biomarkers to identify the disease relapse of AAV.

## 1 Introduction

Antineutrophil cytoplasmic antibody (ANCA)-associated vasculitis (AAV) refers to a potentially fatal, relatively rare group of autoimmune diseases that may affect multiple systems. AAV causes inflammation and damage to small- and medium-sized vessels and is characterized by multisystem organ involvement with alternating periods of relapse and remission (1, 2). Beyond that, a smouldering progress during apparently clinically silent phases often develops (3). The complicated clinical course of vasculitis makes the study of biomarkers a difficult task.

To assess the disease activity of AAV and to customize treatments, the Birmingham vasculitis activity score (BVAS), vasculitis damage index, and five factor score (FFS) are commonly used in clinical practice (4–9). The evaluation of disease through clinical symptoms often causes delayed results, and the item-scoring system is complicated. Thus, reliable biomarkers that can objectively reflect the disease progression and activity for prompt, precise treatments must be identified. Existing biomarkers such as ANCA titer and nonspecific inflammatory markers such as C-reactive protein (CRP) and erythrocyte sedimentation rate (ESR) are insufficient to assess AAV activities. Several cytokines, chemokines, soluble receptors, and markers of microvascular damage are under intensive research for their potential as new biomarkers of AAV (10–12). Some of these circulating immune mediators are elevated in patients with highly active AAV and decline after treatment, distinguishing active AAV from remission better than conventional markers (10–12). However, studies regarding the interdependent relationship between these immune-response mediators are limited. Determining whether these molecules form a complete pathogenic signaling cascade, as well as identifying biomarkers that can promptly predict disease activity and new therapeutic targets for AAV, are crucial.

The present study aimed to determine the profiles of plasma immune response cytokines in patients with AAV, explore the major signaling pathway involved in AAV pathogenesis by analyzing the relationship among these cytokines, and identify the potential biomarkers that can immediately indicate disease activity.

## 2 Methods

### 2.1 Study populations

Patients with AAV who visited the First Affiliated Hospital of Zhengzhou University and fulfilled the Chapel Hill Consensus Conference definitions (13) were consecutively enrolled between January 2022 and June 2023. The exclusion criteria were as follows: the basic information of patients was incomplete, or the patients had other autoimmune diseases, infection or other serious comorbidities. Healthy controls (HCs) were matched with AAV patients for gender and age, and had no autoimmune disease, severe allergic disorder, malignancy, or infection. Clinical data of patients were recorded. BVAS was used to evaluate AAV activity, and FFS was used to evaluate prognosis at diagnosis. Patients’ demographics are shown in **Table 1**. This study was approved by the institutional review board of the First Affiliated Hospital of Zhengzhou University. Informed consent was obtained from all study participants.

**Table 1:** Demographic and clinical characteristics of the AAV patients.

Characteristic	AAV patients (n=59)
Age at time of sample, M (IQR)	68 (58-72)
Age at time of diagnosis, M (IQR)	66 (57-70)
Male, n (%)	30 (50.8%)
**Clinical features**	
ENT	12 (20.3%)
Lung	40 (67.8%)
Cardiovascular	5 (8.5%)
Kidney	20 (33.9%)
Nervous system	9 (15.3%)
**Laboratory features**	
ESR (mm/h), M (IQR)	34 (13-65.5) (n=58)
CRP (mg/L), M (IQR)	7.17 (1.50-63.51)
MPO-positive (n, %)	50 (84.7%)
PR3-positive (n, %)	9 (15.3%)
**BVAS (2003),** M (IQR)	5 (3-9)
**FFS (2011),** M (IQR)	1 (1-2)
**Treatment, n (%)**	
Prednisone	56 (94.9%)
CTX	24 (40.7%)
RTX	16 (27.1%)
AZA	3 (5.1%)
MTX	1 (1.7%)
PLEX	1 (1.7%)
IVIG	5 (8.5%)

AAV, antineutrophil cytoplasmic antibody-associated vasculitis; ENT, ear-nose-throat; M, median; IQR, inter-quartile range; SD, standard deviation; ESR, erythrocyte sedimentation rate; CRP, Creactive protein; MPO, myeloperoxidase; PR3, proteinase 3; BVAS, Birmingham Vasculitis Activity Score; FFS, Five-Factor Score; CTX, Cyclophosphamide; RTX, Rituximab; AZA, Azathioprine; MTX, Methotrexate; PLEX, Plasmapheresis; IVIG, intravenous immunoglobulin.

### 2.2 Sample acquisition and detection of cytokines

Peripheral blood (2–5 mL) was collected into EDTA tubes. The blood samples were centrifuged at 1000 ×g at 4°C for 10 min, and plasma samples were stored as aliquots at -80°C until assays were performed. Before testing, the samples were thawed, vortexed, and centrifuged at 10 000 ×g for 5 min to remove particulates. Plasma levels of 80 cytokines were measured using ProcartaPlex™ Human Immune Response Panel 80-Plex kit (ThermoFisher). Use Luminex™200™ for readings. All assays were performed according to the manufacturer’s instructions. Results were analyzed using ProcartaPlex™ Analyst 1.0.

### 2.3 Statistical analysis

Continuous data were described as the mean ± standard deviation or median (interquartile range), and frequency (percentage) was used for categorical data. The Mann–Whitney U test was used to determine differences in cytokine levels between patients with AAV and HCs, as well as between disease activity and disease remission. The significantly different cytokine concentrations are shown as a heat map. Data were normalized (first, 1 was added to all result data, and then the logarithm of log10 was taken. Then, Z-score was performed on each result according to the group to exclude the influence of extreme values on the results). The heat map was drawn on the Bioladder website (https://www.bioladder.cn/). Binary logistic-regression analysis was used to screen for cytokines associated with the risk of AAV. Spearman’s rank correlation coefficient was used to examine correlations of cytokine levels with ESR, CRP, BVAS, FFS, MPO, and PR3. Correlation and matrix analyses were further performed using the R packages “psych” and “Hmisc,” and results were visualized using the R package “corrplot.” Differences between the two groups of paired samples showed a skewed distribution using Wilcoxon sign-rank test. The difference value normal distribution used a paired-design t-test. All statistical analyses were conducted using IBM SPSS Statistics (version 25.0). Figures were drawn in GraphPad Prism (version 9.5.1).

### 2.4 Bioinformatic analysis

T-distributed stochastic neighbor embedding (t-SNE) analysis using the R package “Rtsne” was visualized with the R package “ggplot2.” Results of Gene Ontology (GO) and Kyoto Encyclopedia of Genes and Genomes (KEGG) enrichment analyses were obtained from the GO (http://www.geneontology.org/) and KEGG (http://www.kegg.jp/kegg/kegg1.html) databases. The protein–protein interaction (PPI) network was derived using the STRING Protein Interaction network database (https://cn.string-db.org/). The PPI network was visualized with Cytoscape software (version 3.10.1).

### 2.5 Receiver operating characteristic analysis

ROC analysis for cytokines was conducted to determine the optimal cut-off points and sensitivity and specificity to differentiate patients with AAV from HCs, as well as patients in remission from those in relapse. The data were analyzed, and graphs were created using GraphPad Prism (version 9.5.1). The flow chart of this study is shown in **Supplementary Figure S1**.

## 3 Results

### 3.1 Demographics and clinical features of patients with AAV

The baseline clinical features of the participants are shown in **Table 1**. A total of 59 patients with AAV were enrolled (50.8% male). The median age at time of diagnosis was 66 (IQR, 44–66) years. Serologic ANCA types were available in all patients, including 50 (84.7%) myeloperoxidase-ANCA (MPO-ANCA)-positive patients and 9 (15.3%) proteinase 3-ANCA (PR3-ANCA)-positive patients. Among the 59 patients with AAV, 48 (81.4%) had active disease, and 11 (18.6%) were in remission. The median BVAS for all patients was 5 (IQR, 3–9). The median FFS for all patients was 1 (IQR, 1–2).

### 3.2 Profiles of cytokines in patients with AAV

**Figure 1:**
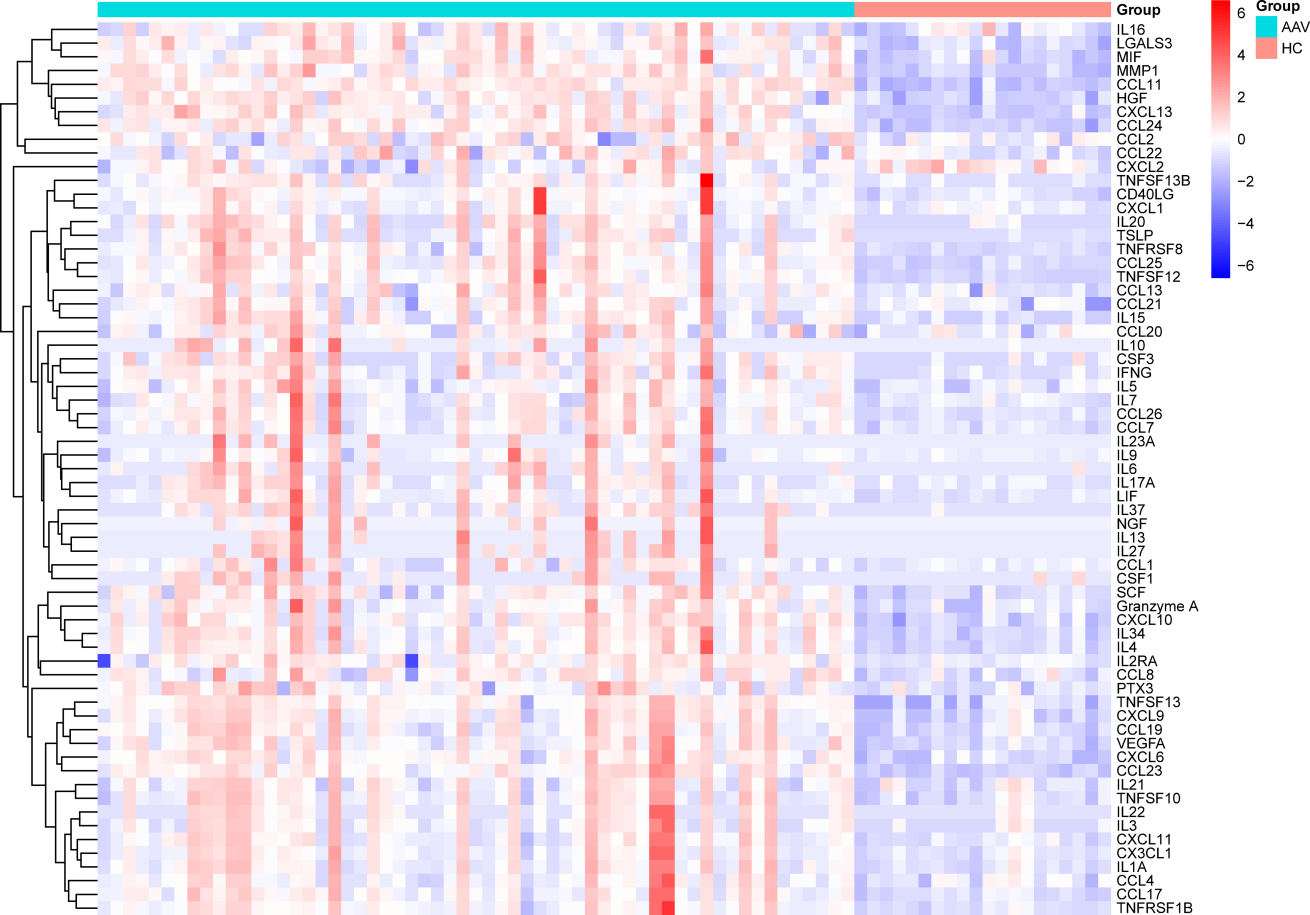
Clustering heatmap of differently expressed cytokines.

Differences in the cytokine levels of patients with AAV and HCs were compared. The expression levels of cytokines in plasma samples from the AAV group and HCs are shown in **Supplementary Table S1**. A total of 65 cytokines significantly differed between the two groups (*p*<0.05). All differentially expressed cytokines except CXCL2 were higher in the AAV group than in HCs. **Figure 1** shows the heat map of 65 cytokine levels with significant differences in expression between the two groups. Subsequently, binary logistic-regression analysis was performed to explore the effect of 65 cytokines on the risk of AAV. Results demonstrated that 43 cytokines had a statistically significant effect on the risk of AAV (**Supplementary Table S2**).

### 3.3 t-SNE

**Figure 2:**
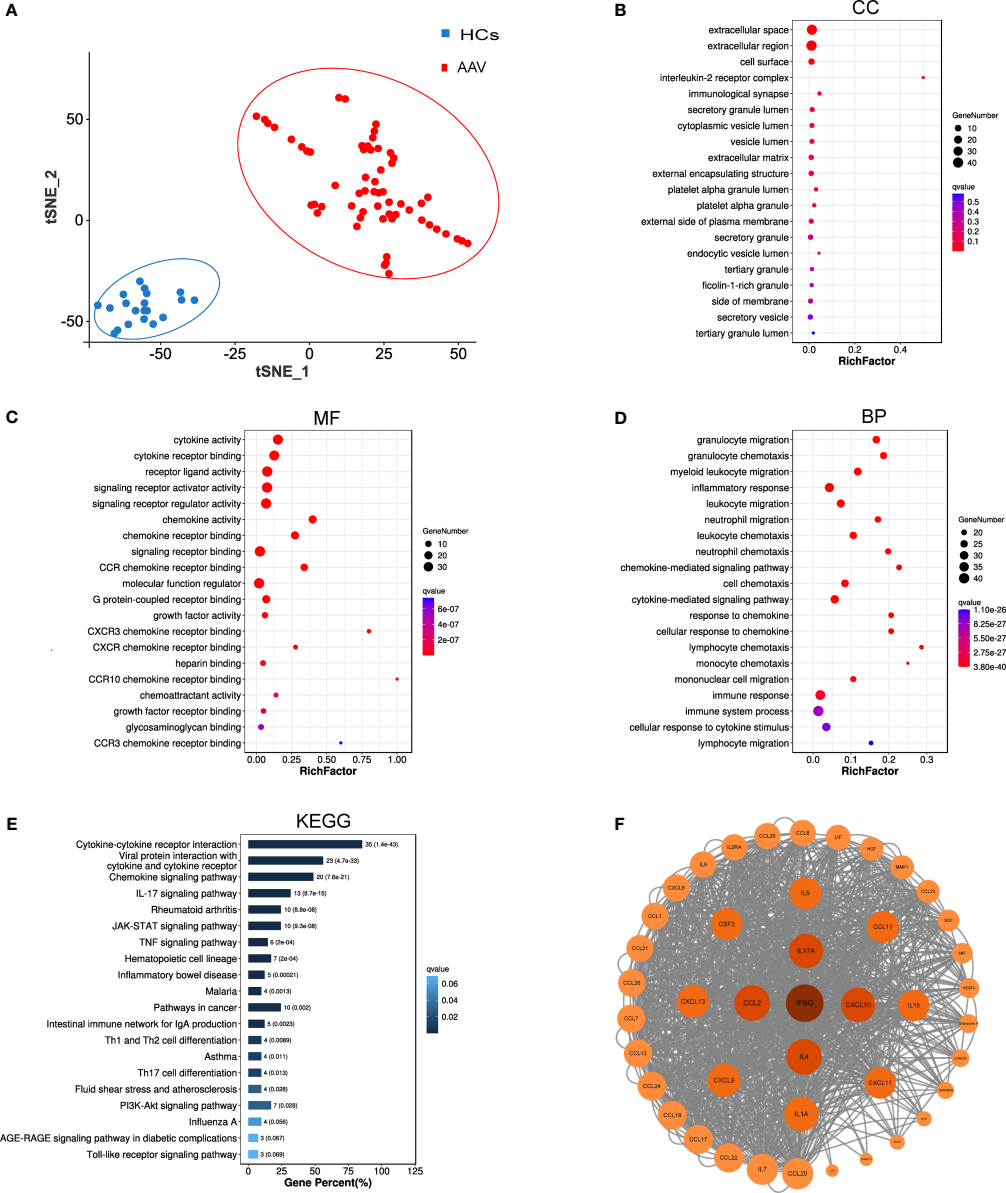
Bioinformatic analysis results of 43 key cytokines. **(A)** T-SNE analysis of 43 different cytokine levels in AAV patients (AAV, n = 59) and healthy controls (HCs, n = 20). **(B–D)** GO analysis of 43 cytokines, cellular components (CC), molecular functions (MF), biological processes (BP). **(E)** KEGG pathway analysis of 43 cytokines. **(F)** Protein-protein interaction network of the 43 cytokines. Each circle reresents one cvtokine, and the darker the color, the larger the circle, and the closer the location to the center of the circle, the more core the cytokine is in the analysis of the PPI network.

If the 43 risk-associated cytokines screened were specific to patients with AAV, those with AAV and HCs would be well separated by t-SNE analysis characterized by 43 cytokine plasma concentration levels. **Figure 2A** shows that the t-SNE analysis of these 43 cytokines may effectively differentiate 59 patients with AAV from 20 HCs.

### 3.4 Functional enrichment analysis and PPI network

GO and KEGG analyses were conducted in the 43 cytokines associated with the risk of AAV. **Figures 2B–E** show the top 20 results of the GO and KEGG analyses of q value (*p* value after multiple corrections) in ascending order. The GO analysis in **Figure 2B** shows that the most related cellular components were extracellular space. **Figure 2C** shows that the most related molecular functions were cytokine activity. The top 20 pathways enriched by GO analysis were primarily involved in biological processes such as inflammatory response, immune response and system process, and chemotaxis and migration of neutrophils, lymphocytes, and monocytes (**Figure 2D**). The pathways of KEGG analysis primarily included cytokine–cytokine receptor interaction, viral protein interaction with cytokine and cytokine receptor, chemokine signaling pathway, IL-17 signaling pathway, rheumatoid arthritis, JAK-STAT signaling pathway, TNF signaling pathway, and other signaling pathways. To gain further insights into PPIs between differentially expressed cytokines, PPI network-interaction diagrams were constructed (**Figure 2F**).

### 3.5 ROC analysis of 43 cytokines

**Figure 3:**
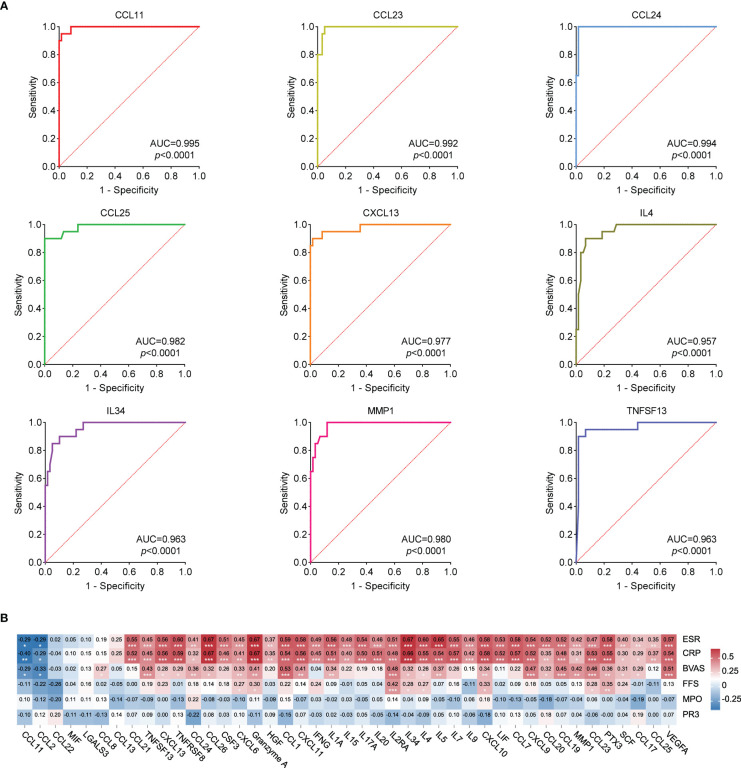
**(A)** ROC curves that differentiate AAV patients from healthy controls (Top 9 best AUC values). The area under curve (AUC) values indicate cytokines can be served as predictor biomarkers for AAV diagnosis. **(B)** Heat map of correlation analysis between 43 cytokine levels and ESR, CRP, FFS, BVAS, MPO and PR3. Red represents the positive correlation, blue represents the negative correlation, and the number represents the correlation coefficient. * stands for significance (*p<0.05, **p<0.01, ***p<0.001).

ROC analysis of the 43 cytokines was conducted to distinguish patients with AAV from HCs. Results showed these 43 cytokines were effective in differentiating patients with AAV from HCs (**Supplementary Table S3**). **Figure 3A** shows the ROC curve of the top 9 cytokines with the largest AUC distinguishing AAV from HC (CCL11: AUC=0.995, CCL23: AUC=0.992, CCL24: AUC=0.994, CCL25: AUC=0.982, CXCL13: AUC=0.977, IL4: AUC=0.957, IL34: AUC=0.963, MMP1: AUC=0.980, and TNFSF13: AUC=0.963).

### 3.6 Correlation between the levels of 43 cytokines and clinical characteristics in patients with AAV

Spearman correlation analysis was conducted between the levels of 43 cytokines and ESR, CRP, BVAS, FFS, MPO, and PR3 in patients with AAV. Results of the correlation analysis among the 43 cytokines are shown in **Supplementary Figure S2**. **Figure 3B** shows that the levels of 36 cytokines were significantly positively correlated with ESR and CRP (CCL21, TNFSF13, CXCL13, TNFRSF8, CCL24, CCL26, CSF3, CXCL6, Granzyme A, HGF, CCL1, CXCL11, IFNG, IL1A, IL15, IL17A, IL20, IL2RA, IL34, IL4, IL5, IL7, IL9, CXCL10, LIF, CCL7, CXCL9, CCL20, CCL19, MMP1, CCL23, PTX3, SCF, CCL17, CCL25, and VEGFA), whereas the levels of 2 cytokines were significantly negatively correlated with ESR and CRP (CCL11 and CCL2). The levels of 25 cytokines were significantly positively correlated with BVAS (CCL8, TNFSF13, CXCL13, TNFRSF8, CCL24, CCL26, CXCL6, Granzyme A, CCL1, CXCL11, IL1A, IL2RA, IL34, IL4, IL5, CXCL10, CXCL9, CCL20, CCL19, MMP1, CCL23, PTX3, SCF, CCL17, and VEGFA), whereas the levels of 2 cytokines were significantly negatively correlated with BVAS (CCL11 and CCL2). These results suggested that some of these cytokines were closely correlated with the disease activity.

### 3.7 Profile of 43 cytokines in paired samples before and after treatment remission

**Figure 4:**
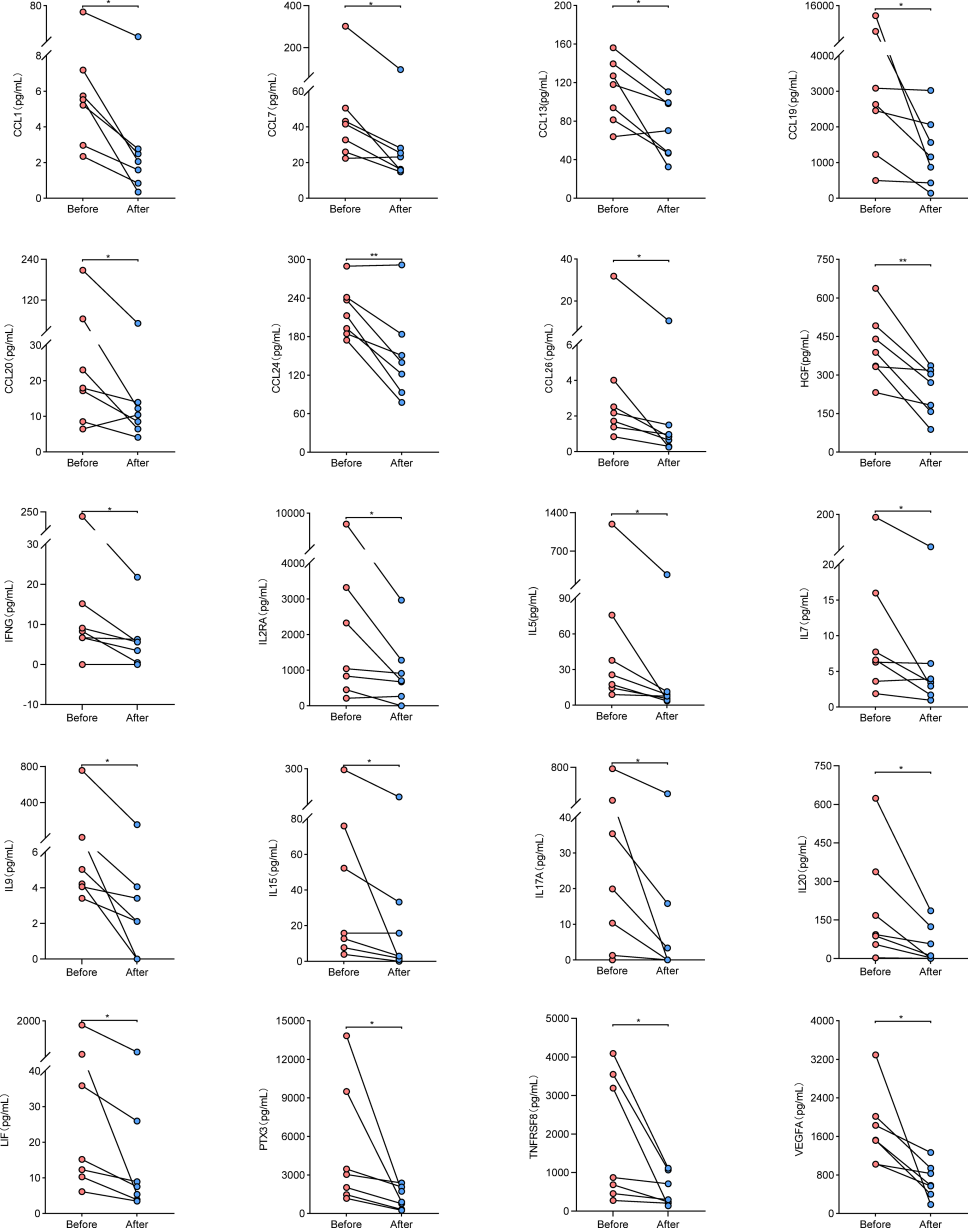
Cytokine levels in paired samples from AAV patients before and after treatment remission. Symbols represent individual subjects. * stands for significance (*p<0.05, **p<0.01).

To determine whether the cytokine levels varied with disease activity, we randomly screened seven patients with active disease and measured the levels of the 43 cytokines again in the same way after their disease went into remission. These cytokine levels were compared before and after treatment remission. Results showed that the levels of 42 cytokines other than CCL11 decreased after treatment remission (**Supplementary Table S4**), among which 20 significantly decreased compared with before treatment remission (CCL1, CCL7, CCL13, CCL19, CCL20, CCL24, CCL26, HGF, IFNG, IL2RA, IL5, IL7, IL9, IL15, IL17A, IL20, LIF, PTX3, TNFRSF8, and VEGFA), as shown in **Figure 4**. These findings confirmed that the cytokine levels changed with disease activity.

### 3.8 Comparison of cytokines in disease initial group, remission group, and relapse group

**Figure 5:**
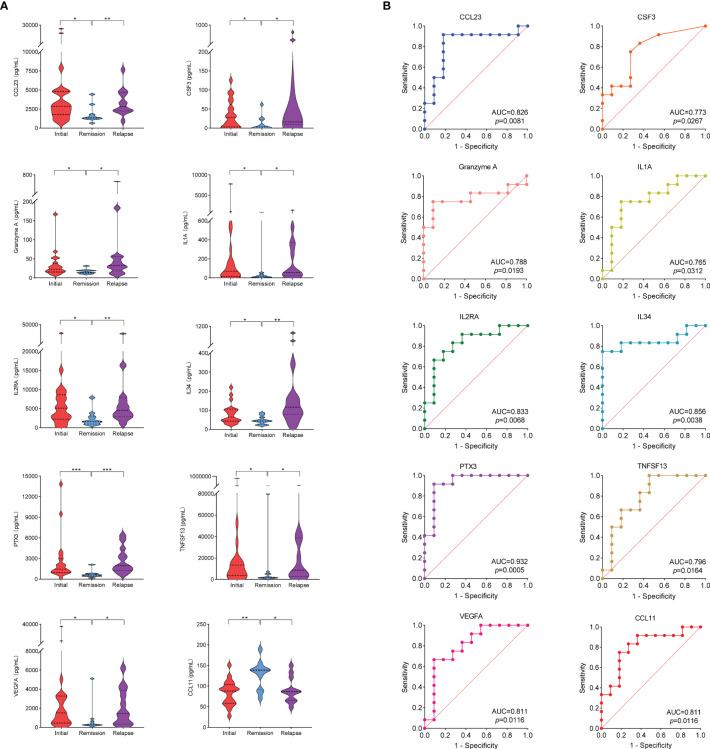
**(A)** Cytokine levels in initial group, remission group and relapse group. * stands for significance (*p<0.05, **p<0.01, ***p<0.001). **(B)** ROC curves that differentiate relapse patients from remission. The area under curve (AUC) values indicate cytokines can be served as predictor biomarkers for AAV disease relapse.

Due to the different active states of AAV disease, the treatment options also varied. According to 2022 EULAR guidelines (14), patients were divided into active (presence of typical signs, symptoms, or other features of active AAV), remission (absence of typical signs, symptoms, or other features of active AAV with or without immunosuppressive therapy), and relapse (recurrence of active AAV after a period of remission) groups. Patients in active stage may be treated with high-dose glucocorticoids and immunosuppressants. Thus, to avoid the influence of powerful drug therapy on cytokine levels, we screened 21 patients with active stage before first medication for the initial group, 11 patients in remission for the remission group, and 12 patients with relapse before treatment adjustment for the relapse group. The clinical characteristics of the initial, remission and relapse patients with AAV are shown in **Table 2**. Mann–Whitney U test was used to compare the cytokine levels among different groups. Among them, the levels of 17 cytokines in the initial group were significantly higher than those in the remission group (CCL1, CCL8, CCL19, CCL20, CCL23, CCL24, CSF3, CXCL9, Granzyme A, IL1A, IL2RA, IL5, IL34, MMP1, PTX3, TNFSF13, and VEGFA), whereas 2 cytokines in the initial treatment group were significantly lower than those in the remission group (CCL2 and CCL11). The level of 15 cytokines in the relapse group was significantly higher than that in the remission group (CCL23, CSF3, CXCL10, CXCL11, CXCL13, Granzyme A, IFNG, IL1A, IL2RA, IL4, IL7, IL34, PTX3, TNFSF13, and VEGFA), whereas 1 cytokine in the relapse group was significantly lower than that in the remission group (CCL11) (**Supplementary Table S5**). **Figure 5A** shows that 9 cytokine levels significantly decreased in the remission group compared with the other two groups (CCL23, CSF3, Granzyme A, IL1A, IL2RA, IL34, PTX3, TNFSF13, and VEGFA), whereas 1 cytokine level significantly increased in the remission group compared with the other two groups (CCL11).

**Table 2:** Demographic and clinical characteristics of the Initial, Remission and Relapse patients with AAV.

Characteristic	Initial (n=21)	Remission (n=11)	Relapse (n=12)
Age at time of sample, M (IQR)	67 (53-70)	60 (54-69)	72 (66-77)
Male, n (%)	10 (47.6%)	6 (54.5%)	6 (50.0%)
**Clinical features**			
ANCA-ILD, n (%)	11 (52.4%)	5 (45.5%)	5 (41.7%)
ANCA-GN, n(%)	7 (33.3%)	3 (27.3%)	3 (25.0%)
**Laboratory features**			
ESR (mm/h), Mean±SD	54±35	14±10	65±32
CRP (mg/L), M (IQR)	13 (3-64)	2 (2-5)	72 (32-90)
MPO-positive (n, %)	19 (90.5%)	10 (90.9%)	10 (83.3%)
PR3-positive (n, %)	2 (9.5%)	1 (9.1%)	2 (16.7%)
**BVAS (2003),** M (IQR)	10 (6-19)	2 (0-3)	4 (2-7)
**FFS (2011),** M (IQR)	3 (2-3)	2 (1-2)	3 (2-3)

AAV, antineutrophil cytoplasmic antibody-associated vasculitis; mean, mean value; SD, standard deviation; M, median; IQR, inter-quartile range; ANCA, Antineutrophil Cytoplasmic Antibody; ANCA-ILD, ANCA-associated interstitial lung disease; ANCA-GN, ANCA -associated glomerulonephritis; ESR, erythrocyte sedimentation rate; CRP, Creactive protein; MPO, myeloperoxidase; PR3, proteinase 3; BVAS, Birmingham Vasculitis Activity Score; FFS, Five-Factor Score.

### 3.9 Identification of plasma biomarkers to indicate disease relapse

To evaluate the performance of the 10 cytokines that significantly differed in the remission group compared with the other two groups as biomarkers of disease relapse, ROC analysis was performed (**Figure 5B**). Elevated levels of the 9 cytokines suggested a risk of disease relapse (CCL23: AUC=0.826, CSF3: AUC=0.773, Granzyme A: AUC=0.788, IL1A: AUC=0.765, IL2RA: AUC=0.833, IL34: AUC=0.856, PTX3: AUC=0.932, TNFSF13: AUC=0.796, and VEGFA: AUC=0.811). The AUC of CCL11 in disease relapse and remission was 0.811 (*p*=0.0116). However, unlike the other 9 cytokines, the high levels of CCL11 were negatively correlated with the risk of disease relapse (**Supplementary Table S6**).

## 4 Discussion

AAV may affect many systems throughout the body and has protean clinical manifestations. However, the prompt, correct diagnosis and the evaluation of disease activity pose a tremendous challenge to rheumatology communities. Reliable biomarkers can help reveal the pathogenesis of disease and can also reflect disease progression and relapse, thereby guiding early diagnosis and treatment. A systematic approach to identifying the biomarkers for assess AAV activity remains an unmet medical need. In the present study, Human Immune Response Panel 80-Plex was used to detect cytokines in the plasma of patients with AAV and HCs. A group of cytokines were found to be closely correlated with disease activity.

The cytokines differentially expressed in AAV and HC groups mainly included chemokines, interleukins, receptors and ligands, interferon, proteases, growth factors, colony-stimulating factors, and so on. These cytokines interact with one another and contribute to the occurrence of AAV disease through pathogenic pathways, such as cytokine–cytokine receptor interaction, viral protein interaction with cytokine and cytokine receptor, chemokine signaling pathway, IL-17 signaling pathway, rheumatoid arthritis, JAK-STAT signaling pathway, TNF signaling pathway, and other signaling pathways. Severalsignaling pathway of differentially expressed gene enrichment between ANCA-associated glomerulonephritis (ANCA-GN) patients and HCs (GSE108113 and GSE104948 data sets, microdissected glomerular tissue) was consistent with our results, and multiple differentially expressed genes were significantly elevated in ANCA-GN patients, concordant with our findings (15). The blocking drugs related to these pathogenic pathways are extensively used in rheumatic or autoimmune diseases diseases. For example, TNF signaling pathway blockers are used to treat rheumatoid arthritis, ankylosing spondylitis, psoriasis, Crohn’s disease, etc. (16). JAK pathway inhibitors are used to treat rheumatoid arthritis, psoriatic arthritis, ankylosing spondylitis, etc. (17). IL-17 inhibitors are used in the treatment of psoriasis and ankylosing spondylitis (18, 19). The key biomarkers involved in these signaling pathways may serve as therapeutic targets for patients with AAV.

The levels of 25 cytokines were significantly positively correlated with BVAS, whereas those of 2 cytokines were significantly negatively correlated with BVAS. Nine cytokines were identified to effectively reflect disease activity (CCL23, CSF3, Granzyme A, IL1A, IL2RA, IL34, PTX3, TNFSF13, and VEGFA). The cytokine levels were significantly higher in patients with AAV than in HCs and significantly increased during the disease active phase and decreased during the disease remission phase.

PTX3 as a standalone biomarker achieved an area under the ROC curve of 0.932 for identifying AAV relapse, which performed best in these cytokines. PTX3 levels were higher during active disease compared with inactive disease. It was positively correlated with ESR, CRP, BVAS and FFS (PTX3 and ESR; r=0.58; *p*<0.001; PTX3 and CRP; r=0.55; *p*<0.001; PTX3 and BVAS; r=0.36; *p*<0.01; PTX3 and FFS; r=0.35; *p*<0.01). PTX3 is a typical acute phase protein primarily produced by myeloid (20). Vascular inflammation is a major driver of PTX3 elevation (21). PTX3 is also selectively stored in neutrophil-specific particles and is released rapidly in response to recognizing inflammatory signals (20, 22). PTX3 can also be localized in neutrophil extracellular traps (NETs), and PTX3-deficient neutrophils have defective phagocytic activity. NETs play an important role in the pathogenesis of AAV (23–25). Antibodies directed against PTX3 have been detected in various autoimmune diseases, especially in SLE and AAV (22). Additionally, anti-PTX3 antibodies share some characteristics with ANCAs, and the prevalence of anti-PTX3 antibodies appears to be high in ANCA-negative AAVs, which can constitute a useful marker in these situations (22). The increase in PTX3 can effectively identify disease relapse in a timely manner. The potential pathogenic effects of PTX3 and its antibodies and whether they can serve as therapeutic targets requires further study.

IL34, IL2RA, CCL23, and VEGFA can effectively identify disease relapse in patients with AAV (AUC>0.8; *p*<0.05). Although available evidence is insufficient to explain the involvement of these cytokines in the occurrence of AAV diseases, they have a complex interrelationship with various autoimmune diseases and immune cells. The main function of IL34 is to promote monocyte survival, proliferation, and differentiation to macrophages (26). IL2RA is expressed in activated T cells, regulatory T cells, activated B cells, activated monocytes, and NK cells (27, 28). CCL23 and VEGFA plays important roles in the formation of blood vessels, and a large amount of CCL23 and VEGFA induces the proliferation and migration of vascular endothelial cells in the later period of injury, thereby promoting angiogenesis (29, 30). VEGFA and CCL23 were elevated in AAV patients, these phenomena become more obvious during the disease active phase, which may be related to the vascular repair involved in the injury in AAV.

TNFSF13 is closely related to the survival of B cells and maintains the homeostasis of B cells by regulating the function and survival rate of B cells stimulated by antigen (31). TNFSF13B is also a key survival factor for peripheral B cells. TACI, BCMA, and BAFF-R, receptors for TNFSF13 and TNFSF13B are expressed on B cells (32). Our study found that TNFSF13 and TNFSF13B were significantly elevated in patients with AAV. Among them, TNFSF13 was closely related to the disease activity of AAV, and its high level can effectively identify disease relapse (AUC=0.796; *p*<0.05). Belimumab, a monoclonal antibody against TNFSF13B, is used to treat SLE in humans. Similarly, the potential therapeutic value of TNFSF13 blockers in patients with AAV should not be underestimated, and more research is warranted.

In human peripheral blood tests, MPO-ANCA is significantly higher after CSF3 stimulation. In mice, CSF3 administration can aggravate ANCA vasculitis by activating neutrophils *in vivo* (33). In our study, CSF3 levels were significantly higher in patients with AAV than in HCs and increased with disease activity. These data suggested that CSF3, which increased in patient plasma, may indicate disease activity and also play an important role in AAV exacerbation. A recent study has demonstrated that CSF2 increased the ability of ANCA-stimulated monocytes to generate IL1-β and to promote Th17 effector cell polarization. Thus, CSF2 is a crucial pathological factor modulating monocyte proinflammatory functions and, in turn, Th17 cell polarization in AAV patients (34). However, the function and role of CSF3 in AAV pathogenesis remains to be explored.

Previous studies have found the presence of IL1A in kidney biopsy specimens from (ANCA-GN) patients (35). In an *in vitro* culture of glomerular cells in patients with rapidly progressing crescentic glomerulonephritis, a gradual increase in IL1A production over time has been found (36). Our study showed that IL1A was significantly higher in the plasma of patients with AAV than HCs and varied with disease activity, significantly increased during disease activity and decreasing significantly after disease remission. This result indicated that the elevation of IL1A was closely related with the occurrence, activity, and target organ involvement of AAV disease. Lan et al. (37) successfully treated an established experimental crescentic glomemlonephritis by administering an IL1A receptor antagonist. Although the mechanism of action of IL1A is unclear, IL1A can effectively identify disease relapse. Thus, inhibiting the rise of IL1A can delay disease progression, which has great value for research on this disease.

Interestingly, the levels of two other chemokines, CCL2 and CCL11, were also significantly higher in patients with AAV than in HCs. Unlike the other cytokines, they were negatively correlated with ESR, CRP, and BVAS and were lower in the active disease than in remission. These results suggested a negative relationship between CCL2, CCL11 and disease activity. CCL11 can also effectively distinguish between patients in remission and those with relapse (AUC=0.811; *p*<0.05). Disease relapse is associated with low CCL11 expression in AAV patients. Similar findings were observed in patients with Behcet’s disease, and low disease activity was associated with a higher expression of CCL11 (38). Several studies have shown that urine CCL2 levels in patients with active AAV are significantly higher than patients in remission and healthy controls (39–42), and elevated levels of urine CCL2 are associated with the involvement of important target organs of the disease and poor prognosis (41). However, there is still a lack of valid evidence on whether CCL2 levels in the serum of patients with AAV are associated with disease activity (40, 43). Herjan and colleagues have shown that the IL17A treatment of airway smooth muscle cells can reduce CCL11 expression in a mouse model of allergic asthma (44). The altered expression of CCL11 in patients with AAV may indicate the activation of the IL-17 signaling pathway. Given that IL17A has an inhibitory effect on CCL11 production, it increases and CCL11 decreases at the active phase of the disease. Our study found a negative correlation between CCL11 and IL17A (r=-0.158, *p*=0.233) (**Supplementary Figure S2**). Whether CCL11 merely passively changed with the IL17A level or actively increased through some unknown mechanism to reduce disease activity and prevent disease progression requires further exploration.

In conclusion, our study proved the validity of previous research results from different perspectives. We found some cytokines that together constituted a complex interaction system and played a crucial role in the pathogenesis of AAV. Improved our understanding of the signaling pathway in AAV pathogenesis may facilitate the development of novel therapeutic targets. We also found several biomarkers in this group of cytokines closely related to disease activity, which can effectively identify the relapse of AAV patients and assist in their timely treatment.

However, our study had several limitations. First, the sample size was relatively small, indicating a proneness to deviation in data interpretation. Second, comparison with other autoimmune diseases to determine which cytokines were specific to patients with AAV was not performed. Third, the cytokines measured were all in peripheral blood plasma, and their actual situation in target tissues and organs cannot be detected.

## 5 Conclusions

We found a group of cytokines that may be involved in the pathogenesis of AAV. In addition to the diagnostic value of AAV, some cytokines were closely related to disease activity, among which PTX3, IL34, IL2RA, CCL23, and VEGFA can be used as effective biomarkers to identify AAV disease relapse. Thus, our findings supported further investigation of the cytokines as biomarkers of disease activity and potential therapeutic targets for AAV development.

## Data availability statement

The raw data supporting the conclusions of this article will be made available by the authors, without undue reservation.

## Ethics statement

The studies involving humans were approved by the institutional review board of the First Affiliated Hospital of Zhengzhou University. The studies were conducted in accordance with the local legislation and institutional requirements. The human samples used in this study were acquired from primarily isolated as part of your previous study for which ethical approval was obtained. Written informed consent for participation was not required from the participants or the participants’ legal guardians/next of kin in accordance with the national legislation and institutional requirements.

## Author contributions

WH: Conceptualization, Data curation, Formal analysis, Investigation, Methodology, Software, Supervision, Validation, Visualization, Writing – original draft. WL: Conceptualization, Formal analysis, Funding acquisition, Investigation, Methodology, Project administration, Resources, Supervision, Validation, Visualization, Writing – review & editing. XW: Investigation, Writing – review & editing. FD: Investigation, Writing – review & editing. PL: Investigation, Writing – review & editing. XZ: Investigation, Writing – review & editing. RL: Investigation, Writing – review & editing. TL: Writing – review & editing. LZ: Investigation, Resources, Supervision, Writing – review & editing. SL: Conceptualization, Formal analysis, Project administration, Resources, Supervision, Validation, Writing – review & editing.

## Supplementary material

Supplementary Figure 1 Flow chart of this study.

Supplementary Figure 2 Correlation heat map of 43cytokine levels in patients with AAV. Red represents positive correlation, blue represents negative correlation, white represents no correlation, numbers represent the correlation coeficients, and * represents significant correlation. (**p*<0.05, ***p*<0.01, ****p*<0.001, *****p*<0.0001).
