# Supplementary figures and images for: Profiles of cytokines in patients with antineutrophil cytoplasmic antibody-associated vasculitis
Source: Front Immunol. 2024 Jul 23;15:1428044. doi: 10.3389/fimmu.2024.1428044 (PMC11300338; doi:10.3389/fimmu.2024.1428044)

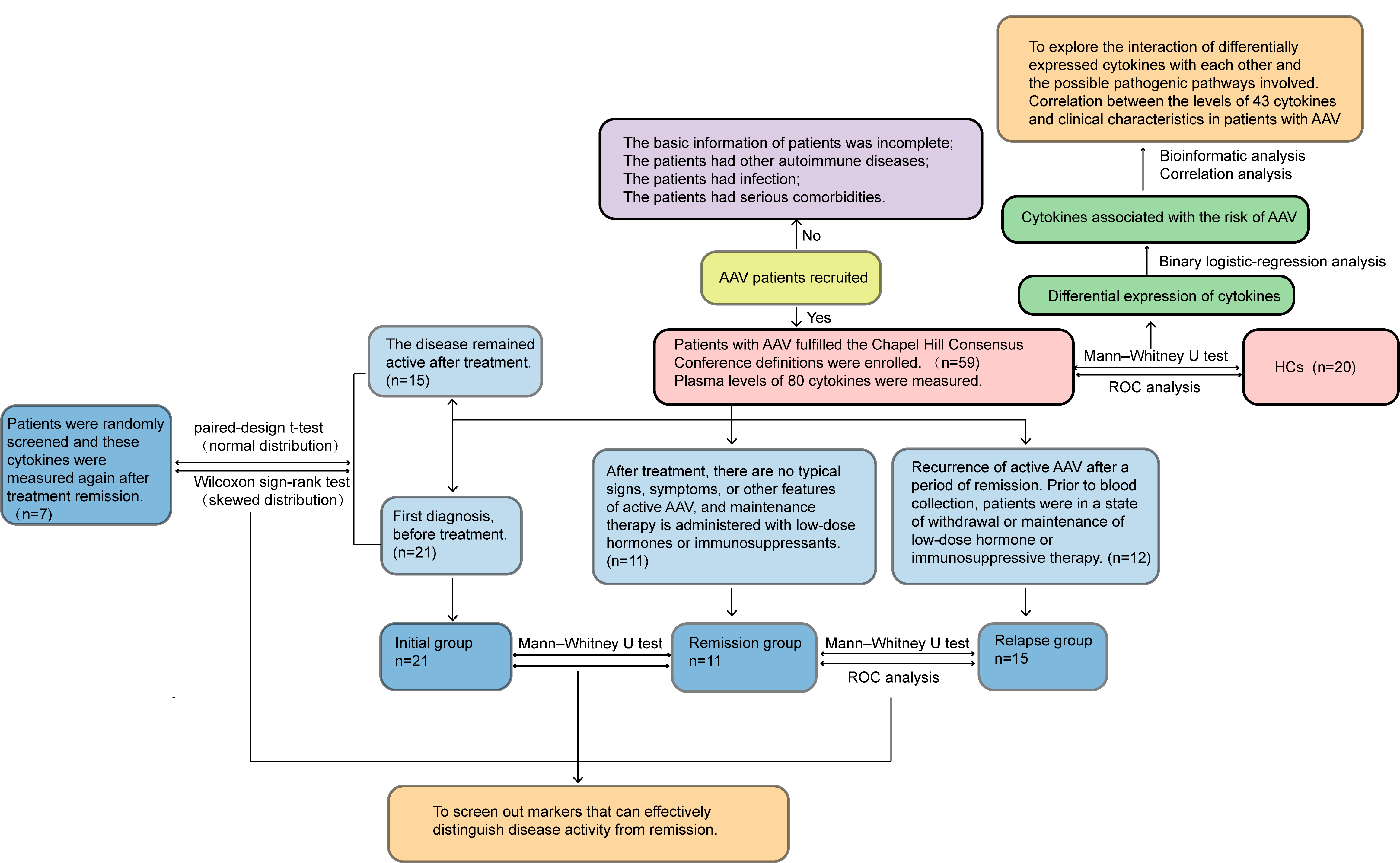

Supplement: Supplementary Figure 1 — Flow chart of this study. [file Image_1.tif]
