# Supplementary material for: Profiles of cytokines in patients with antineutrophil cytoplasmic antibody-associated vasculitis
Source: Front Immunol. 2024 Jul 23;15:1428044. doi: 10.3389/fimmu.2024.1428044 (PMC11300338; doi:10.3389/fimmu.2024.1428044)
Supplement: Supplementary file 3 [file Table_1.docx]

**Supplementary Table S1.** Comparison of cytokine concentration between AAV group and HC group

| Cytokine | M (P_25_, P_75_) (pg/mL) | | d (95% CI) | Mann Whitney U test | |
| --- | --- | --- | --- | --- | --- |
|  | AAV | HC |  | Z-value | *p-* value |
| CCL1 | 3 (2, 7) | 2 (2, 2) | 1 (0, 3) | 2.39 | **0.016** |
| CCL2 | 51 (21, 94) | 34 (17, 48) | 18 (2, 42) | 2.15 | **0.031** |
| CCL3 | 16 (5, 39) | 28 (3, 82) | -7 (-27, 5) | -1.12 | 0.267 |
| CCL4 | 133 (79, 216) | 86 (48, 152) | 45 (8, 92) | 2.32 | **0.020** |
| CCL7 | 25 (17, 41) | 14 (12, 18) | 11 (7, 18) | 4.66 | **＜0.0001** |
| CCL8 | 14 (8, 22) | 5 (4, 8) | 7 (4, 11) | 4.24 | **＜0.0001** |
| CCL11 | 94 (71, 134) | 15 (11, 22) | 80 (68, 98) | 6.58 | **＜0.0001** |
| CCL13 | 79 (56, 118) | 46 (41, 61) | 30 (16, 46) | 4.09 | **＜0.0001** |
| CCL17 | 49 (21, 109) | 9 (6, 18) | 39 (20, 62) | 5.34 | **＜0.0001** |
| CCL19 | 765 (267, 3104) | 89 (30, 250) | 662 (264, 1246) | 4.90 | **＜0.0001** |
| CCL20 | 14 (4, 23) | 7 (4, 10) | 5 (0, 11) | 2.20 | **0.028** |
| CCL21 | 141 (60, 316) | 62 (26, 93) | 76 (32, 146) | 3.48 | **＜0.001** |
| CCL22 | 191 (110, 321) | 109 (74, 160) | 89 (33, 171) | 3.04 | **0.002** |
| CCL23 | 2242 (1353, 3324) | 421 (324, 585) | 1769 (1243, 2389) | 6.55 | **＜0.0001** |
| CCL24 | 160 (133, 231) | 52 (42, 64) | 111 (88, 152) | 6.57 | **＜0.0001** |
| CCL25 | 152 (57, 860) | 12 (7, 15) | 140 (68, 371) | 6.41 | **＜0.0001** |
| CCL26 | 1 (1, 3) | 0 (0, 0) | 1 (1, 2) | 5.01 | **＜0.0001** |
| CD40LG | 174 (94, 562) | 22 (5, 40) | 151 (90, 277) | 5.91 | **＜0.0001** |
| CSF1 | 0 (0, 10) | 0 (0, 0) | 0 (0, 0) | 2.23 | **0.024** |
| CSF2 | 0 (0, 3) | 0 (0, 0) | 0 (0, 0) | 1.64 | 0.094 |
| CSF3 | 8 (0, 34) | 0 (0, 2) | 8 (3, 17) | 3.80 | **＜0.0001** |
| CX3CL1 | 16 (6, 89) | 3 (2, 6) | 10 (4, 26) | 4.26 | **＜0.0001** |
| CXCL1 | 11 (7, 25) | 8 (4, 12) | 5 (1, 9) | 2.63 | **0.008** |
| CXCL2 | 19 (10, 29) | 29 (17, 77) | -12 (-25, -2) | -2.37 | **0.017** |
| CXCL5 | 179 (80, 289) | 288 (112, 450) | -95 (-197, 0) | -1.95 | 0.051 |
| CXCL6 | 83 (68, 126) | 28 (18, 37) | 56 (44, 72) | 5.77 | **＜0.0001** |
| CXCL8 | 74 (22, 147) | 38 (17, 109) | 24 (-4, 64) | 1.62 | 0.107 |
| CXCL9 | 198 (40, 648) | 3 (0, 17) | 182 (67, 378) | 5.39 | **＜0.0001** |
| CXCL10 | 109 (61, 180) | 28 (19, 43) | 72 (42, 109) | 5.67 | **＜0.0001** |
| CXCL11 | 99 (37, 334) | 23 (19, 47) | 64 (22, 112) | 4.28 | **＜0.0001** |
| CXCL13 | 596 (317, 1306) | 70 (60, 92) | 516 (313, 669) | 6.35 | **＜0.0001** |
| FGF2 | 2 (0, 22) | 17 (0, 37) | -2 (-16, 0) | -1.36 | 0.175 |
| Granzyme A | 19 (13, 31) | 8 (5, 14) | 11 (6, 17) | 4.48 | **＜0.0001** |
| Granzyme B | 19 (2, 48) | 20 (8, 25) | 0 (-7, 14) | -0.28 | 0.786 |
| HGF | 288 (153, 440) | 38 (31, 64) | 236 (162, 297) | 6.02 | **＜0.0001** |
| IFNA1 | 0 (0, 0) | 0 (0, 0) | 0 (0, 0) | 1.60 | 0.182 |
| IFNG | 5 (1, 9) | 0 (0, 1) | 4 (2, 6) | 4.43 | **＜0.0001** |
| IL1A | 29 (11, 123) | 7 (3, 13) | 21 (8, 45) | 4.40 | **＜0.0001** |
| IL1B | 0 (0, 6) | 0 (0, 3) | 0 (0, 0) | 0.43 | 0.669 |
| IL2 | 7 (0, 17) | 7 (3, 12) | 0 (-3, 5) | 0.11 | 0.912 |
| IL2RA | 3625 (1634, 6967) | 835 (491, 1478) | 2549 (1488, 3779) | 4.60 | **＜0.0001** |
| IL3 | 54 (6, 1747) | 0 (0, 20) | 47 (10, 265) | 3.75 | **＜0.001** |
| IL4 | 88 (59, 173) | 25 (19, 31) | 65 (45, 101) | 6.08 | **＜0.0001** |
| IL5 | 11 (6, 22) | 4 (1, 7) | 7 (3, 13) | 3.84 | **＜0.0001** |
| IL6 | 0 (0, 27) | 0 (0, 0) | 0 (0, 6) | 2.97 | **0.003** |
| IL7 | 4 (2, 10) | 2 (2, 3) | 2 (1, 4) | 3.35 | **＜0.001** |
| IL9 | 5 (3, 7） | 3 (3, 4) | 1 (0, 3) | 2.89 | **0.003** |
| IL10 | 0 (0, 3) | 0 (0, 0) | 0 (0, 0) | 2.58 | **0.009** |
| IL12 | 0 (0, 3) | 2 (0, 2) | 0 (0, 0) | -0.67 | 0.508 |
| IL13 | 0 (0, 0) | 0 (0, 0) | 0 (0, 0) | 2.37 | **0.023** |
| IL15 | 10 (4, 28) | 0 (0, 2) | 9 (5, 16) | 5.06 | **＜0.0001** |
| IL16 | 234 (103, 342) | 111 (52, 219) | 84 (21, 170) | 2.64 | **0.008** |
| IL17A | 4 (0, 20) | 0 (0, 2) | 3 (0, 8) | 3.00 | **0.002** |
| IL18 | 37 (15, 70) | 28 (23, 32) | 8 (-7, 21) | 1.05 | 0.296 |
| IL20 | 23 (2, 110) | 0 (0, 0) | 19 (7, 68) | 4.48 | **＜0.0001** |
| IL21 | 109 (23, 1561) | 9 (1, 143) | 84 (16, 359) | 3.04 | **0.002** |
| IL22 | 27 (0, 1131) | 0 (0, 0) | 22 (0, 150) | 3.28 | **＜0.001** |
| IL23A | 0 (0, 0) | 0 (0, 0) | 0 (0, 0) | 2.37 | **0.024** |
| IL27 | 0 (0, 13) | 0 (0, 0) | 0 (0, 0) | 2.67 | **0.009** |
| IL31 | 0 (0, 0) | 0 (0, 0) | 0 (0, 0) | 1.72 | 0.136 |
| IL34 | 50 (35, 101) | 15 (13, 19) | 36 (25, 68) | 6.12 | **＜0.0001** |
| IL37 | 3 (0, 12) | 0 (0, 0) | 3 (0, 6) | 3.87 | **＜0.0001** |
| LGALS3 | 39119 (25688, 57857) | 13190 (7176, 18145) | 25990 (18823, 33505) | 5.89 | **＜0.0001** |
| LIF | 9 (5, 20) | 4 (3, 6) | 5 (3, 11) | 5.08 | **＜0.0001** |
| MIF | 53 (39, 81) | 19 (13, 24) | 33 (25, 44) | 5.72 | **＜0.0001** |
| MMP1 | 404 (204, 753) | 39 (25, 69) | 355 (246, 530) | 6.39 | **＜0.0001** |
| NGF | 0 (0, 0) | 0 (0, 0) | 0 (0, 0) | 2.27 | **0.031** |
| PTX3 | 1176 (661, 2125) | 532 (395, 769) | 541 (258, 1059) | 3.87 | **＜0.0001** |
| SCF | 15 (7, 31) | 2 (2, 3) | 12 (6, 20) | 5.24 | **＜0.0001** |
| TNFRSF1B | 152 (91, 304) | 39 (28, 52) | 104 (70, 164) | 5.65 | **＜0.0001** |
| TNFRSF8 | 607 (280, 1410) | 86 (58, 128) | 514 (301, 784) | 5.58 | **＜0.0001** |
| TNFSF10 | 54 (13, 626) | 8 (1, 17) | 40 (13, 193) | 4.30 | **＜0.0001** |
| TNFSF12 | 2635 (1702, 4794) | 354 (296, 440) | 2259 (1797, 3139) | 6.65 | **＜0.0001** |
| TNFSF13 | 5342 (2051, 15743) | 135 (0, 403) | 4799 (2551, 7583) | 6.16 | **＜0.0001** |
| TNFSF13B | 10 (2, 22) | 0 (0, 1) | 8 (3, 15) | 4.59 | **＜0.0001** |
| TNF-α | 0 (0, 7) | 5 (0, 16) | 0 (-5, 0) | -1.88 | 0.059 |
| TNF-β | 0 (0, 0) | 0 (0, 0) | 0 (0, 0) | 2.06 | 0.057 |
| TREM1 | 161 (0, 1082) | 161 (0, 542) | 0 (0, 396) | 1.14 | 0.259 |
| TSLP | 7 (0, 70) | 0 (0, 0) | 7 (2, 16) | 4.60 | **＜0.0001** |
| VEGFA | 700 (288, 2017) | 149 (88, 202) | 547 (270, 1187) | 5.60 | **＜0.0001** |

Values highlighted in bold represent statistically signifificant *p*-values (*p*< 0.05).
