# Supplementary material for: Profiles of cytokines in patients with antineutrophil cytoplasmic antibody-associated vasculitis
Source: Front Immunol. 2024 Jul 23;15:1428044. doi: 10.3389/fimmu.2024.1428044 (PMC11300338; doi:10.3389/fimmu.2024.1428044)
Supplement: Supplementary file 4 [file Table_2.docx]

**Supplementary Table S2.** The cytokines related to the pathogenesis of AAV

| Cytokine | OR (95% CI) | *p* |
| --- | --- | --- |
| CCL1 | 1.524 (1.049-2.215) | **0.027** |
| CCL2 | 1.021 (1.003-1.039) | **0.021** |
| CCL4 | 1.008 (1-1.016) | 0.051 |
| CCL7 | 1.234 (1.092-1.395) | **0.001** |
| CCL8 | 1.236 (1.085-1.407) | **0.001** |
| CCL11 | 1.177 (1.069-1.296) | **0.001** |
| CCL13 | 1.047 (1.017-1.077) | **0.002** |
| CCL17 | 1.123 (1.045-1.207) | **0.002** |
| CCL19 | 1.003 (1.001-1.005) | **0.013** |
| CCL20 | 1.065 (1.003-1.131) | **0.040** |
| CCL21 | 1.012 (1.002-1.023) | **0.018** |
| CCL22 | 1.01 (1.003-1.017) | **0.007** |
| CCL23 | 1.016 (1.001-1.032) | **0.036** |
| CCL24 | 1.216 (1.052-1.404) | **0.008** |
| CCL25 | 1.158 (1.063-1.261) | **0.001** |
| CCL26 | 18.647 (3.386-102.7) | **0.001** |
| CD40LG | 1 (1-1) | 0.664 |
| CSF1 | 1.109 (0.968-1.272) | 0.136 |
| CSF3 | 1.104 (1.012-1.204) | **0.025** |
| CX3CL1 | 1 (1-1) | 0.618 |
| CXCL1 | 1 (1-1.001) | 0.692 |
| CXCL2 | 0.986 (0.973-1) | 0.050 |
| CXCL6 | 1.082 (1.044-1.121) | **0.000** |
| CXCL9 | 1.013 (1.003-1.023) | **0.013** |
| CXCL10 | 1.081 (1.036-1.128) | **0.000** |
| CXCL11 | 1.025 (1.006-1.045) | **0.012** |
| CXCL13 | 1.019 (1.008-1.03) | **0.001** |
| Granzyme A | 1.196 (1.073-1.334) | **0.001** |
| HGF | 1.038 (1.015-1.062) | **0.001** |
| IFNG | 1.985 (1.226-3.214) | **0.005** |
| IL1A | 1.04 (1.001-1.081) | **0.046** |
| IL2RA | 1.001 (1-1.002) | **0.001** |
| IL3 | 1 (1-1) | 0.800 |
| IL4 | 1.124 (1.054-1.199) | **0.000** |
| IL5 | 1.174 (1.048-1.315) | **0.006** |
| IL6 | 1.092 (0.975-1.224) | 0.129 |
| IL7 | 1.494 (1.104-2.021) | **0.009** |
| IL9 | 1.417 (1.024-1.962) | **0.036** |
| IL10 | 2.535 (0.541-11.872) | 0.238 |
| IL13 | 370.079(0- ) | 0.993 |
| IL15 | 1.605 (1.204-2.139) | **0.001** |
| IL16 | 1.003 (1-1.007) | 0.064 |
| IL17A | 1.316 (1.024-1.69) | **0.032** |
| IL20 | 1.095 (1.007-1.192) | **0.034** |
| IL21 | 1.001 (1-1.002) | 0.115 |
| IL22 | 1 (1-1) | 0.665 |
| IL23A | 617.237 (0-3.75E+135) | 0.967 |
| IL27 | 3.838 (0-3.25E+54) | 0.983 |
| IL34 | 1.234 (1.095-1.392) | **0.001** |
| IL37 | 1.923 (0.976-3.786) | 0.059 |
| LGALS3 | 1 (1-1) | **0.000** |
| LIF | 2.042 (1.309-3.185) | **0.002** |
| MIF | 1.137 (1.07-1.209) | **0.000** |
| MMP1 | 1.051 (1.017-1.085) | **0.003** |
| NGF | 670.008 (0-8.53E+148) | 0.970 |
| PTX3 | 1.002 (1-1.003) | **0.011** |
| SCF | 1.43 (1.15-1.778) | **0.001** |
| TNFRSF1B | 1 (1-1) | 0.737 |
| TNFRSF8 | 1.016 (1.006-1.026) | **0.002** |
| TNFSF10 | 1.007 (0.998-1.015) | 0.113 |
| TNFSF12 | 4.499 (0.175-115.451) | 0.364 |
| TNFSF13 | 1.002 (1.001-1.003) | **0.001** |
| TNFSF13B | 1 (1-1) | 0.760 |
| TSLP | 2.656 (0.98-7.201) | 0.055 |
| VEGFA | 1.009 (1.003-1.015) | **0.003** |

Values highlighted in bold represent statistically signifificant *p*-values (*p*<0.05).
