# Supplementary material for: Profiles of cytokines in patients with antineutrophil cytoplasmic antibody-associated vasculitis
Source: Front Immunol. 2024 Jul 23;15:1428044. doi: 10.3389/fimmu.2024.1428044 (PMC11300338; doi:10.3389/fimmu.2024.1428044)
Supplement: Supplementary file 5 [file Table_3.docx]

**Supplementary Table S3.** Statistical data of ROC curve comparisons of different parameters in identifying AAV patients

| Cytokines | AUC (95% CI) | Youden index | Cut-off value（pg/mL） | Sensitivity | Specificity | *p*-value |
| --- | --- | --- | --- | --- | --- | --- |
| CCL1 | 0.68 (0.57,0.79) | 0.46 | 3.47 | 0.46 | 1 | **0.017** |
| CCL2 | 0.661 (0.54,0.78) | 0.37 | 72.08 | 0.37 | 1 | **0.032** |
| CCL7 | 0.85 (0.77,0.93) | 0.71 | 20.47 | 0.71 | 1 | **<0.0001** |
| CCL8 | 0.819 (0.72,0.92) | 0.56 | 7.97 | 0.76 | 0.8 | **<0.0001** |
| CCL11 | 0.995 (0.98,1) | 0.93 | 35.66 | 0.98 | 0.95 | **<0.0001** |
| CCL13 | 0.808 (0.71,0.91) | 0.54 | 67.43 | 0.64 | 0.9 | **<0.0001** |
| CCL17 | 0.902 (0.83,0.97) | 0.64 | 26.76 | 0.69 | 0.95 | **<0.0001** |
| CCL19 | 0.869 (0.78,0.96) | 0.58 | 207.61 | 0.83 | 0.75 | **<0.0001** |
| CCL20 | 0.664 (0.54,0.79) | 0.36 | 11.35 | 0.56 | 0.8 | **0.029** |
| CCL21 | 0.762 (0.65,0.87) | 0.49 | 120.83 | 0.59 | 0.9 | **<0.001** |
| CCL22 | 0.728 (0.62,0.84) | 0.49 | 228.92 | 0.49 | 1 | **0.002** |
| CCL23 | 0.992 (0.98,1) | 0.95 | 754.27 | 0.95 | 1 | **<0.0001** |
| CCL24 | 0.994 (0.98,1) | 0.98 | 79.29 | 0.98 | 1 | **<0.0001** |
| CCL25 | 0.982 (0.95,1) | 0.9 | 27.19 | 1 | 0.9 | **<0.0001** |
| CCL26 | 0.876 (0.8,0.95) | 0.68 | 0.62 | 0.78 | 0.9 | **<0.0001** |
| CSF3 | 0.776 (0.67,0.88) | 0.49 | 4.56 | 0.59 | 0.9 | **<0.001** |
| CXCL6 | 0.934 (0.88,0.99) | 0.77 | 47.3 | 0.92 | 0.85 | **<0.0001** |
| CXCL9 | 0.905 (0.83,0.98) | 0.72 | 18.05 | 0.92 | 0.8 | **<0.0001** |
| CXCL10 | 0.926 (0.86,0.99) | 0.73 | 46.64 | 0.88 | 0.85 | **<0.0001** |
| CXCL11 | 0.822 (0.72,0.92) | 0.53 | 30.57 | 0.83 | 0.7 | **<0.0001** |
| CXCL13 | 0.977 (0.94,1) | 0.88 | 104.58 | 0.98 | 0.9 | **<0.0001** |
| Granzyme A | 0.836 (0.74,0.94) | 0.51 | 10.67 | 0.86 | 0.65 | **<0.0001** |
| HGF | 0.952 (0.91,1) | 0.85 | 108.16 | 0.85 | 1 | **<0.0001** |
| IFNG | 0.831 (0.74,0.92) | 0.61 | 1.09 | 0.76 | 0.85 | **<0.0001** |
| IL1A | 0.831 (0.73,0.93) | 0.53 | 18.37 | 0.63 | 0.9 | **<0.0001** |
| IL2RA | 0.846 (0.76,0.93) | 0.68 | 1922.9 | 0.73 | 0.95 | **<0.0001** |
| IL4 | 0.957 (0.91,1) | 0.83 | 37.83 | 0.93 | 0.9 | **<0.0001** |
| IL5 | 0.788 (0.68,0.89) | 0.46 | 8.28 | 0.61 | 0.85 | **<0.001** |
| IL7 | 0.752 (0.65,0.86) | 0.53 | 3.37 | 0.68 | 0.85 | **<0.001** |
| IL9 | 0.717 (0.61,0.83) | 0.53 | 4.14 | 0.58 | 0.95 | **0.004** |
| IL15 | 0.877 (0.8,0.95) | 0.71 | 3.31 | 0.81 | 0.9 | **<0.0001** |
| IL17A | 0.72 (0.61,0.83) | 0.53 | 2.49 | 0.58 | 0.95 | **0.003** |
| IL20 | 0.828 (0.74,0.92) | 0.58 | 0.48 | 0.78 | 0.8 | **<0.0001** |
| IL34 | 0.96 (0.92,1) | 0.8 | 20.67 | 0.95 | 0.85 | **<0.0001** |
| LGALS3 | 0.942 (0.89,0.99) | 0.75 | 24045.52 | 0.8 | 0.95 | **<0.0001** |
| LIF | 0.881 (0.8,0.96) | 0.56 | 5.03 | 0.86 | 0.7 | **<0.0001** |
| MIF | 0.93 (0.85,1) | 0.8 | 28.81 | 0.95 | 0.85 | **<0.0001** |
| MMP1 | 0.98 (0.96,1) | 0.88 | 121.86 | 0.88 | 1 | **<0.0001** |
| PTX3 | 0.791 (0.68,0.9) | 0.56 | 804.57 | 0.71 | 0.85 | **<0.001** |
| SCF | 0.894 (0.82,0.97) | 0.73 | 6.15 | 0.78 | 0.95 | **<0.0001** |
| TNFRSF8 | 0.919 (0.86,0.98) | 0.86 | 185.86 | 0.86 | 1 | **<0.0001** |
| TNFSF13 | 0.963 (0.91,1) | 0.88 | 707.44 | 0.98 | 0.9 | **<0.0001** |
| VEGFA | 0.921 (0.86,0.99) | 0.75 | 229.74 | 0.9 | 0.85 | **<0.0001** |

Values highlighted in bold represent statistically signifificant P values (*P* < 0.05)
