# Supplementary material for: Profiles of cytokines in patients with antineutrophil cytoplasmic antibody-associated vasculitis
Source: Front Immunol. 2024 Jul 23;15:1428044. doi: 10.3389/fimmu.2024.1428044 (PMC11300338; doi:10.3389/fimmu.2024.1428044)
Supplement: Supplementary file 6 [file Table_4.docx]

Supplementary Table S4-1. Comparison of cytokine levels between paired active disease and remission samples

| Cytokines | M(P25,P75) (pg/mL) | | Paired Samples Wilcoxon Signed Rank Test | |
| --- | --- | --- | --- | --- |
|  | Active | Remission | Z-value | *p*- value |
| CCL1 | 6(3,7) | 2(1,3) | 2.37 | **0.016** |
| CCL7 | 42(26,51) | 23(16,28) | 2.2 | **0.031** |
| CCL19 | 2633(1228,7324) | 1159(430,2064) | 2.37 | **0.016** |
| CCL20 | 18(9,65) | 10(6,14) | 2.2 | **0.031** |
| CCL26 | 2(1,4) | 1(0,2) | 2.37 | **0.016** |
| CSF3 | 70(0,105) | 20(3,46) | 1.78 | 0.094 |
| CXCL9 | 576(319,2004) | 314(42,470) | 1.52 | 0.156 |
| CXCL10 | 75(57,162) | 47(36,202) | 0.34 | 0.813 |
| Granzyme A | 24(13,27) | 17(7,45) | 1.18 | 0.297 |
| IFNG | 8(7,15) | 6(0,6) | 2.2 | **0.031** |
| IL1A | 115(58,338) | 54(7,109) | 1.35 | 0.219 |
| IL2RA | 1041(450,3320) | 706(269,1282) | 2.2 | **0.031** |
| IL5 | 26(15,76) | 8(4,11) | 2.37 | **0.016** |
| IL7 | 7(4,16) | 3(2,6) | 2.03 | **0.047** |
| IL9 | 5(4,12) | 2(0,4) | 2.37 | **0.016** |
| IL15 | 16(8,76) | 3(1,33) | 2.2 | **0.031** |
| IL17A | 20(1,96) | 0(0,16) | 2.2 | **0.031** |
| LIF | 15(10,166) | 8(4,26) | 2.37 | **0.016** |
| PTX3 | 3044(1464,9497) | 890(323,2062) | 2.37 | **0.016** |
| TNFRSF8 | 871(456,3550) | 297(208,1069) | 2.37 | **0.016** |
| VEGFA | 1524(1038,2017) | 585(403,940) | 2.37 | **0.016** |

Values highlighted in bold represent statistically signifificant *p*-values (*p*< 0.05).

Supplementary Table S4-2. Comparison of cytokine levels between paired active disease and remission samples

| Cytokines | Mean±SD | | d(95%CI) | Paired t-test | |
| --- | --- | --- | --- | --- | --- |
|  | Active | Remission |  | t-value | *p*-value |
| CCL2 | 32±24 | 32±18 | 0(-29, 29) | 0.01 | 0.996 |
| CCL8 | 21±22 | 15±10 | 6(-10, 21) | 0.89 | 0.406 |
| CCL11 | 95±39 | 117±32 | -22(-57, 14) | -1.50 | 0.184 |
| CCL13 | 111±33 | 72±31 | 39(11, 68) | 3.38 | **0.015** |
| CCL17 | 205±211 | 69±46 | 136(-59, 331) | 1.71 | 0.138 |
| CCL21 | 785±792 | 224±279 | 561(-18, 1139) | 2.37 | 0.055 |
| CCL22 | 264±91 | 166±115 | 99(-14, 212) | 2.14 | 0.076 |
| CCL23 | 2633±568 | 1919±380 | 714(-33, 462) | 2.34 | 0.058 |
| CCL24 | 219±40 | 151±71 | 68(29, 106) | 4.27 | **0.005** |
| CCL25 | 1640±1831 | 411±554 | 1228(-32, 2489) | 2.39 | 0.054 |
| CXCL6 | 105±38 | 80±21 | 25(-11, 62) | 1.71 | 0.138 |
| CXCL11 | 348±297 | 141±102 | 207(-105, 518) | 1.62 | 0.155 |
| CXCL13 | 1473±874 | 1316±1051 | 157(-828-1142) | 0.39 | 0.71 |
| HGF | 408±131 | 237±94 | 171(74-268) | 4.33 | **0.005** |
| IL4 | 176±192 | 120±90 | 57(-100-213) | 0.89 | 0.409 |
| IL20 | 195±217 | 54±74 | 141(2-280) | 2.47 | **0.048** |
| IL34 | 109±110 | 72±61 | 37(-66-140) | 0.88 | 0.413 |
| LGALS3 | 34764±17524 | 44198±38146 | -9434(-49197-30329) | -0.58 | 0.583 |
| MIF | 50±14 | 43±18 | 7(-12-27) | 0.89 | 0.41 |
| MMP1 | 614±294 | 534±327 | 79(-81-239) | 1.21 | 0.273 |
| SCF | 22±19 | 13±11 | 9(-5-23) | 1.55 | 0.171 |
| TNFSF13 | 19033±18197 | 6104±4811 | 12930(-4761-30620) | 1.79 | 0.124 |

Values highlighted in bold represent statistically signifificant *p*-values (*p*< 0.05).
