# Supplementary material for: Profiles of cytokines in patients with antineutrophil cytoplasmic antibody-associated vasculitis
Source: Front Immunol. 2024 Jul 23;15:1428044. doi: 10.3389/fimmu.2024.1428044 (PMC11300338; doi:10.3389/fimmu.2024.1428044)
Supplement: Supplementary file 7 [file Table_5.docx]

**Supplementary Table S5. Comparison of cytokine concentration between Initial, Remission and Relapse group**

| Cytokines | M (P_25_, P_75_) (pg/mL) | | | Mann Whitney U test | | | |
| --- | --- | --- | --- | --- | --- | --- | --- |
|  | Initial | Remission | Relapse | Initial VS Remission | | Relapse VS Remission | |
|  |  |  |  | Z | *p*-value | Z | *p*-value |
| CCL1 | 6 (3, 11) | 2 (1, 3) | 4 (2, 10) | 3.1 | **0.001** | 1.79 | 0.076 |
| CCL2 | 23 (14, 63) | 60 (55, 108) | 48 (18, 133) | 3.23 | **0.001** | 0.98 | 0.347 |
| CCL7 | 31 (22, 50) | 22 (16, 31) | 33 (18, 190) | 1.67 | 0.098 | 1.36 | 0.184 |
| CCL8 | 15 (8, 23) | 12 (6, 14) | 19 (8, 29) | 2.04 | **0.042** | 1.60 | 0.118 |
| CCL11 | 88 (58, 104) | 138 (91, 141) | 87 (66, 113) | 2.96 | **0.002** | 2.52 | **0.011** |
| CCL13 | 95 (56, 137) | 72 (55, 85) | 77 (60, 121) | 0.97 | 0.347 | 0.62 | 0.566 |
| CCL17 | 70 (22, 186) | 28 (15, 52) | 61 (30, 238) | 1.77 | 0.079 | 1.42 | 0.169 |
| CCL19 | 2456 (725, 10807) | 312 (228, 798) | 2321 (338, 7848) | 2.72 | **0.006** | 1.60 | 0.118 |
| CCL20 | 19 (12, 34) | 4 (1, 23) | 18 (9, 45) | 2.21 | **0.026** | 1.80 | 0.074 |
| CCL21 | 190 (127, 1281) | 138 (56, 255) | 213 (76, 841) | 1.25 | 0.223 | 1.05 | 0.316 |
| CCL22 | 191 (112, 327) | 275 (110, 424) | 264 (85, 295) | 0.4 | 0.703 | 0.98 | 0.347 |
| CCL23 | 2869 (1789, 4829) | 1353 (1216, 1805) | 2815 (2260, 4660) | 2.36 | **0.017** | 2.65 | **0.007** |
| CCL24 | 227 (161, 282) | 139 (92, 178) | 169 (134, 279) | 2.56 | **0.009** | 1.29 | 0.211 |
| CCL25 | 339 (113, 3019) | 173 (52, 739) | 129 (59, 1864) | 0.99 | 0.331 | 0.37 | 0.729 |
| CCL26 | 2 (1, 4) | 1 (0, 3) | 2 (1, 14) | 1.73 | 0.086 | 1.85 | 0.069 |
| CSF3 | 29 (3, 62) | 3 (0, 23) | 16 (9, 305) | 2.13 | **0.033** | 2.24 | **0.024** |
| CXCL6 | 81 (70, 146) | 66 (39, 85) | 97 (63, 146) | 1.88 | 0.061 | 1.91 | 0.059 |
| CXCL9 | 458 (173, 2264) | 83 (29, 182) | 597 (70, 1900) | 2.36 | **0.017** | 1.88 | 0.062 |
| CXCL10 | 133 (61, 171) | 95 (53, 112) | 176 (90, 248) | 1.49 | 0.144 | 2.46 | **0.013** |
| CXCL11 | 127 (53, 703) | 48 (31, 94) | 319 (61, 670) | 1.88 | 0.060 | 2.09 | **0.037** |
| CXCL13 | 649 (372, 1419) | 317 (187, 611) | 652 (360, 2499) | 1.73 | 0.088 | 2.22 | **0.027** |
| Granzyme A | 23 (16, 37) | 14 (12, 19) | 33 (19, 56) | 1.96 | **0.0495** | 2.34 | **0.018** |
| HGF | 306 (182, 484) | 249 (129, 302) | 365 (148, 636) | 1.69 | 0.096 | 1.35 | 0.190 |
| IFNG | 5 (0, 11) | 2 (0, 7) | 9 (5, 31) | 0.8 | 0.438 | 2.68 | **0.006** |
| IL1A | 71 (18, 408) | 16 (7, 40) | 56 (27, 373) | 2.12 | **0.033** | 2.15 | **0.032** |
| IL2RA | 5137 (2271, 8658) | 1634 (984, 2995) | 4569 (2840, 7872) | 2.24 | **0.025** | 2.71 | **0.006** |
| IL4 | 100 (74, 174) | 84 (43, 151) | 159 (119, 498) | 0.89 | 0.389 | 2.25 | **0.023** |
| IL5 | 18 (10, 36) | 6 (4, 11) | 22 (6, 125) | 2.66 | **0.007** | 1.95 | 0.052 |
| IL7 | 7 (3, 10) | 3 (2, 4) | 5 (3, 29) | 1.85 | 0.066 | 2.19 | **0.028** |
| IL9 | 7 (4, 12) | 5 (3, 7) | 5 (3, 26) | 1.07 | 0.293 | 0.52 | 0.618 |
| IL15 | 17 (7, 57) | 15 (4, 28) | 12 (4, 69) | 1.19 | 0.242 | 0.25 | 0.820 |
| IL17A | 10 (1, 66) | 5 (0, 8) | 12 (1, 262) | 1.18 | 0.246 | 1.15 | 0.261 |
| IL20 | 73 (15, 553) | 11 (0, 90) | 62 (8, 172) | 1.67 | 0.097 | 1.06 | 0.305 |
| IL34 | 64 (43, 106) | 44 (26, 63) | 117 (79, 300) | 2.1 | **0.035** | 2.89 | **0.003** |
| LGALS3 | 37353 (22949, 69974) | 30735 (22241, 41197) | 40437 (36888, 96071) | 0.77 | 0.457 | 1.66 | 0.104 |
| LIF | 12 (6, 52) | 7 (5, 19) | 17 (8, 182) | 1.23 | 0.226 | 1.88 | 0.061 |
| MIF | 53 (38, 81) | 48 (39, 78) | 64 (40, 86) | 0.18 | 0.868 | 0.80 | 0.449 |
| MMP1 | 554 (366, 812) | 220 (85, 380) | 391 (172, 877) | 3.27 | **0.001** | 1.48 | 0.151 |
| PTX3 | 1447 (954, 2982) | 595 (424, 783) | 1948 (1299, 4215) | 3.55 | **<0.001** | 3.51 | **<0.001** |
| SCF | 16 (6, 23) | 7 (6, 29) | 19 (7, 52) | 0.81 | 0.434 | 1.36 | 0.184 |
| TNFRSF8 | 862 (391, 2802) | 488 (238, 766) | 998 (383, 1712) | 1.59 | 0.115 | 1.20 | 0.241 |
| TNFSF13 | 13432 (3648, 43484) | 1567 (1476, 5342) | 8683 (2772, 38645) | 2 | **0.045** | 2.40 | **0.015** |
| VEGFA | 1509 (452, 3284) | 268 (230, 637) | 1469 (334, 3867) | 2.6 | **0.008** | 2.52 | **0.011** |

Values highlighted in bold represent statistically signifificant *P* values (*P* < 0.05)
