# Supplementary material for: Profiles of cytokines in patients with antineutrophil cytoplasmic antibody-associated vasculitis
Source: Front Immunol. 2024 Jul 23;15:1428044. doi: 10.3389/fimmu.2024.1428044 (PMC11300338; doi:10.3389/fimmu.2024.1428044)
Supplement: Supplementary file 8 [file Table_6.docx]

**Supplementary Table S6.** Statistical data of ROC curve comparisons of different parameters in identifying patients with relapse and patients with remission

| Cytokines | AUC (95% CI) | Youden index | Cut-off value (pg/mL) | Sensitivity | Specificity | *p*-value |
| --- | --- | --- | --- | --- | --- | --- |
| CCL23 | 0.826 (0.64,1) | 0.73 | 1981.96 | 0.92 | 0.82 | **0.008** |
| CSF3 | 0.773 (0.58,0.97) | 0.48 | 10.45 | 0.75 | 0.73 | **0.027** |
| Granzyme A | 0.788 (0.58,0.99) | 0.66 | 23.61 | 0.75 | 0.91 | **0.019** |
| IL1A | 0.765 (0.56,0.97) | 0.57 | 42.14 | 0.75 | 0.82 | **0.031** |
| IL2RA | 0.833 (0.66,1) | 0.58 | 3823.08 | 0.67 | 0.91 | **0.007** |
| IL34 | 0.856 (0.68,1) | 0.75 | 87.34 | 0.75 | 1.00 | **0.004** |
| PTX3 | 0.932 (0.82,1) | 0.83 | 879.92 | 0.92 | 0.91 | **<0.0001** |
| TNFSF13 | 0.795 (0.6, 0.99) | 0.55 | 1576.44 | 1.00 | 0.55 | **0.016** |
| VEGFA | 0.811 (0.62,1) | 0.58 | 957.04 | 0.67 | 0.91 | **0.012** |
| CCL11 | 0.811 (0.63, 0.99) | 0.57 | 90.11 | 0.82 | 0.75 | **0.012** |

Values highlighted in bold represent statistically signifificant *P* values (*P* < 0.05)
